# 1‐Methylcyclopropene Inhibits Enzymatic Browning in Lanzhou Lily Bulbs: Involvement of the Ascorbate–Glutathione Cycle in Antioxidant Regulation and Membrane Stabilization

**DOI:** 10.1155/ijfo/8304551

**Published:** 2026-10-19

**Authors:** Jiajia Su, Lihua Duan, Xiaokun Qiu, Jiaxuan Zheng, Ping Li, Runlei Kou, Xia Liu, Qingbin Guo

**Affiliations:** ^1^ State Key Laboratory of Food Nutrition and Safety College of Food Science and Engineering Tianjin University of Science and Technology Tianjin China tust.edu.cn; ^2^ College of Mechanical Engineering Tianjin University of Science and Technology Tianjin China tust.edu.cn; ^3^ Huanxian Food Technology Co. Ltd. Shandong China

**Keywords:** 1-methylcyclopropene (1-MCP), ascorbate–glutathione cycle, enzymatic browning, Lanzhou lily, membrane stability, postharvest senescence, ROS scavenging

## Abstract

To address the issue of scale browning in Lanzhou lily bulbs during postharvest storage, this study evaluated the efficacy of 1‐methylcyclopropene (1‐MCP), a strategy not previously reported for this crop. Treatment with 1.5 *μ*L L^−1^ 1‐MCP effectively alleviated scale browning, as evidenced by reduced respiratory rate, lower weight loss, retained bulb firmness, and intact cellular structure. Mechanistically, likely by interfering with ethylene perception, 1‐MCP enhanced the lily bulbs′ inherent antioxidant capacity by increasing the activities of superoxide dismutase, catalase, ascorbate peroxidase, glutathione reductase, and peroxidase, alongside elevating the contents of ascorbic acid, total phenolics, reduced glutathione, and flavonoids. This treatment downregulated lipoxygenase activity, inhibiting oxidative degradation of membrane unsaturated fatty acids and alleviating membrane lipid peroxidation cascades. It also attenuated superoxide anion generation and diminished hydrogen peroxide accumulation, relieving oxidative stress induced by reactive oxygen species. Significant reductions in malondialdehyde levels and electrolyte leakage further confirmed enhanced cellular membrane stability. Correlation analysis subsequently verified significant intrinsic associations between antioxidant system components, browning inhibition, and quality preservation. Overall, 1‐MCP delayed postharvest browning and improved storage quality in Lanzhou lily bulbs, with the ascorbate–glutathione cycle playing an important contributory role in the antioxidant regulatory network, alongside reducing ROS‐induced membrane lipid peroxidation and preserving membrane integrity. This offers a physiology‐guided strategy for preserving bulb vegetables.

## 1. Introduction

Lanzhou lily (*Lilium davidii* var. *unicolor*), dubbed “vegetable ginseng,” is a member of the genus *Lilium* in the Liliaceae family. As a distinct bulb vegetable endemic to China, it is highly valued for its dual edible and medicinal uses. The bulbs abound in bioactive compounds including polysaccharides, saponins, alkaloids, phenolics, and flavonoids. These compounds confer multiple pharmacological effects, such as antioxidant, antitumor, and hypoglycemic activities [1]. With the steady rise in public health awareness in recent years, market demand for Lanzhou lily has grown. However, the outer scales of postharvest Lanzhou lily are prone to browning, which not only severely impairs their commercial appearance and nutrient retention but also compromises cell membrane integrity, exacerbates redox homeostasis imbalance, weakens the tissue defense barrier, and facilitates the invasion of pathogenic microorganisms [2], leading to significant economic losses.

Enzymatic browning is the primary cause of postharvest browning in Lanzhou lily [3], and it is closely linked to imbalanced reactive oxygen species (ROS) metabolism and impaired cell membrane stability. The peroxidation of unsaturated fatty acids catalyzed by lipoxygenase (LOX) is the pivotal initial step of these sequential changes, and the accumulation of malondialdehyde [4], its terminal metabolite, is a marker of membrane system damage [5]. This process disrupts the cellular compartmentalization structure, leading to the contact between polyphenol oxidase (PPO) and phenolic substrates in vacuoles, thereby initiating enzymatic browning [6]. To cope with oxidative stress, plants have evolved a multilevel ROS scavenging system [7]. The ascorbate–glutathione (AsA‐GSH) cycle plays an important regulatory role in maintaining cellular redox homeostasis. Specifically, this cycle efficiently scavenges hydrogen peroxide (H_2_O_2_) via ascorbate peroxidase (APX) and relies on enzymes such as glutathione reductase (GR) to continuously regenerate antioxidants (AsA and GSH), thereby precisely regulating intracellular H_2_O_2_ levels and overall reducing potential [8]. Enhancing the AsA‐GSH cycle may therefore serve as a key strategy for controlling browning in lily bulbs.

1‐Methylcyclopropene (1‐MCP) is a cyclopropene‐derived gaseous ethylene‐perception inhibitor [9]. Its high‐affinity interaction with ethylene receptors relies on coordination bonding between the *π*‐electron system of its double bond and the Cu^+^ cofactor within receptor proteins [10]. 1‐MCP occupies ethylene‐binding sites on receptor proteins (receptor occupancy), which represents a discrete molecular event that precedes alterations in downstream ethylene‐dependent signal responses [11]. Owing to the intrinsic ring‐strain of the cyclopropene scaffold, 1‐MCP forms considerably more stable receptor‐ligand complexes than does ethylene, with minimal dissociation over the relevant time scale. Upon 1‐MCP binding, conformational rearrangement of the ethylene receptor occurs, maintaining the receptor‐CTR1 complex in an inactive signaling state and preventing the physiological conformational transition triggered by ethylene [12]. This receptor‐level antagonistic effect can effectively retard ripening and senescence in many horticultural commodities [13]. As documented by Blankenship and Dole [11], 1‐MCP exhibits approximately 10‐fold higher binding affinity for ethylene receptors compared with ethylene itself, enabling its preferential association with receptor proteins. In recent decades, 1‐MCP has found widespread application in postharvest fresh‐keeping practices for horticultural fruits and vegetables, and has delivered promising practical effects. On climacteric fruits including apples [14], pears [15] and peaches [16], 1‐MCP exhibits a pronounced alleviative effect on tissue browning, and effectively inhibits postharvest browning in tender rhizome vegetables represented by lotus root [17]. Furthermore, for bulb vegetables such as onions [18], 1‐MCP has been reported to delay postharvest quality deterioration. Nevertheless, no study to date has specifically addressed the effect of 1‐MCP on browning in Lanzhou lily bulbs, a gap that constrains the development of targeted preservation solutions for this specialty bulb vegetable.

Although not typical climacteric organs, lily bulbs are susceptible to postharvest browning linked to cell membrane damage, oxidative stress, and ethylene‐mediated metabolic perturbations [19]. The current study attempts to clarify the regulatory effects of 1‐MCP on alleviating scale browning and maintaining postharvest quality in Lanzhou lily bulbs. We conducted a systematic evaluation of core quality parameters: morphological traits, nutrient components, microstructural integrity, oxidative damage indicators, antioxidant enzyme activities, nonenzymatic antioxidant contents, and browning‐related traits. Furthermore, this study is aimed at elucidating how 1‐MCP exerts its antibrowning effect, with a specific focus on its role in boosting the AsA‐GSH cycle to maintain cell membrane stability and redox homeostasis. Outcomes of this study provide theoretical support and practical technical strategies for mitigating postharvest storage limitations in Lanzhou lily bulbs, thereby enhancing their applicability for fresh consumption and deep processing.

## 2. Materials and Methods

### 2.1. Samples and Postharvest Treatment

The test lily bulbs were collected from Xiguoyuan Village, Qilihe District, Lanzhou City, Gansu Province, China. Immediately after harvest, the bulbs were kept at low temperature and air‐freighted to the State Key Laboratory of Food Nutrition and Safety, College of Food Science and Engineering, Tianjin University of Science and Technology, within 5 h of harvest. Upon arrival, fresh bulbs of uniform size, pest‐free, disease‐free, and free of mechanical damage were selected as experimental materials, and then precooled at 4.0°C for 24 h. Preliminary experiments were conducted prior to the formal experiment to optimize the 1‐MCP concentration range and to confirm its effectiveness in inhibiting browning. For the formal experiment, three independent biological replicates (*n* = 3) were performed using different batches of bulbs. For each biological replicate, approximately 20 kg of bulbs were randomly divided into four groups, with a total weight of approximately 5.0 kg per group. Among them, three treatment groups (1.0, 1.5, and 2.0 *μ*L L^−1^) were fumigated with 1‐MCP for 12 h under sealed conditions at room temperature using commercial 1‐MCP tablets (AnsiP, Lytone, Taiwan, China). According to the product label, the recommended dosage for the standard application concentration is one tablet (1.2 g) per cubic meter; the control group was placed under identical conditions for the same duration but without 1‐MCP exposure. Treated lily bulbs were packaged in 0.03‐mm‐thick perforated polyethylene preservation bags (hole diameter: 2 mm; hole spacing: 50 × 50 mm). The temperature‐controlled chamber was set to 4°C, with a controlled temperature fluctuation range of ±0.5°C, and relative humidity inside the preservation bags was maintained at approximately 80%. Sampling was performed every 12 days during the 60‐day storage.

### 2.2. Color Parameters and Browning Degree (BD)

The color parameters (L*, a*, and b* values) were measured following calibration with the SR‐66 precision color difference meter (Ningbo Ledee Instrument Co. Ltd., China). All lily bulbs were measured at the equatorial region. The BD was calculated in accordance with Equations (1) and (2).

(1) BD=1000.310.172x−/,

(2) X=a∗+1.75L∗5.6453.012L∗+a∗−b∗,

where L* denotes lightness, a* stands for the red‐green axis component, b* corresponds to the yellow‐blue axis component, and BD refers to browning level.

### 2.3. Respiration Rate, Firmness, Weight Loss Rate, and Total Soluble Solids (TSS)

The respiration rate of lily bulbs was measured with a fruit and vegetable respirometer (YT‐GX10, Shandong Tianyan Instrument Co. Ltd., China). Individual lily bulb samples, each weighing 75.0–80.0 g, were placed into the respiratory chamber. Following hermetic sealing, the samples were subjected to respiratory rate testing [20]. The bulbs′ equatorial firmness was quantified with a fruit firmness tester (HPA, Quzhou Apyi Measurement Instrument Co. Ltd., China). Weight loss rate, presented as a percentage, was calculated by dividing the poststorage mass loss of lily bulbs by their initial mass (recorded on Day 0) and multiplying the quotient by 100. A high‐precision digital refractometer (DR101, Beijing Youlai Technology Co. Ltd., China) was employed to measure the TSS content in lily bulbs. Representative, randomly selected bulb samples were processed by peeling outer scales, weighing 20.0 g of peeled scales, and homogenizing the material to a homogeneous pulp with a mechanical homogenizer. Following filtration, the filtrate was assayed.

### 2.4. Microstructure

Microstructural observation was performed following the method of Manzoor et al. [21]. Epidermal tissue of Lanzhou lily scales was examined via a scanning electron microscope (SEM, FE‐1050, NCS Testing Technology Co. Ltd., China). The specific operation is as follows: Lily scales were quickly frozen with liquid nitrogen, then transferred to a lyophilizer (FD‐IA‐50, Shanghai Daluo Scientific Instrument Co. Ltd., China) and freeze‐dried at −50°C; the samples were cut into 3 × 3 × 3 mm uniform cubic blocks, fixed on copper stubs; then the epidermal microstructure of lily samples was observed under the scanning electron microscope at a magnification of 500×.

### 2.5. Assessments of Electrolyte Leakage (EL), MDA, and ROS

The EL assay was performed following the protocol described by Zhu et al. [22]. Ten lily scales were randomly selected and cut into circular thin sections using a 5‐mm diameter punch. The sections were soaked in 20 mL of distilled water for 30 min, and the initial electrical conductivity (P_0_) was measured using a conductivity meter (DS‐1A, Zhejiang Shangke Instrument Export Co. Ltd., China). The samples were then boiled for 10 min to completely release all intracellular electrolytes, and after cooling to room temperature, the final electrical conductivity (P_1_) was measured. EL was calculated using Equation (3).

(3) EL=P1P0×100%,

where P_0_ and P_1_ are the electrical conductivities measured before and after boiling, respectively.

MDA content (*μ*mol g^−1^, FW) was determined using the thiobarbituric acid (TBA) method with 100 g L^−1^ trichloroacetic acid (TCA) and 6.7 g L^−1^ TBA (Sinopharm Chemical Reagent Co. Ltd., China and Sigma‐Aldrich, United States, respectively) according to the method of Kong et al. [23]. H_2_O_2_ content (*μ*mol g^−1^ FW) was assayed using a commercial kit (Grace Biotechnology Co. Ltd., Suzhou, China; Catalog No. G0168W48) according to the manufacturer′s instructions as described by Xie et al. [24]. O_2_^−^· production rate (mmol g^−1^ min^−1^ FW) was measured using a commercial kit (Shanghai Yuanye Bio‐Technology Co. Ltd., China; Catalog No. R30342‐100T) according to the manufacturer′s instructions as described by Wei et al. [25]. Lily bulb samples were randomly selected, outer and inner scales removed, and the remaining scales mixed and chopped thoroughly before quickly weighing 1.0 g; 2.0 mL of O_2_^−^· lysis solution was added and homogenized, with the supernatant collected as O_2_^−^· extract after centrifugation. The extract, O_2_^−^· lysis solution, and hydroxylamine solution were mixed and water‐bathed; the reaction solution was then transferred to the color development solution and mixed well, and then the absorbance was measured.

### 2.6. Assay of Antioxidant Enzyme Activities

Enzyme extract was prepared following Hong et al. [26]. Fresh lily bulbs were randomly selected, outer and inner scales carefully peeled off, and the remaining scales mixed well and chopped. Bulb scale tissue (5.0 g) was quickly weighed, transferred into extraction buffer (5.0 mL), and fully ground. SOD activity was quantified by virtue of 50% suppression of nitroblue tetrazolium photomediated reduction. CAT activity was inferred from absorbance decrease per minute at 240 nm, indicative of H_2_O_2_ degradation. POD activity was assayed using H_2_O_2_ as the substrate and guaiacol as the hydrogen donor, with activity defined as the per‐minute absorbance increase at 470 nm. APX activity was assessed by quantifying ascorbic acid oxidation, with the per‐minute absorbance decline rate at 290 nm measured to indicate activity. Through following the reductive process of oxidized glutathione (GSSG), GR activity was quantified, with activity characterized by the per‐minute absorbance decrease at 340 nm. All enzymatic activities were quantified as units per gram of fresh weight (U g^−1^ FW). The LOX activity was described by Wu et al. [27]. One unit (U) of LOX activity was defined as the absorbance increase per minute at 234 nm in the linoleic acid reaction system under continuous monitoring.

### 2.7. Measurement of Nonenzymatic Antioxidant Substance Content

Ascorbic acid content was determined using the 2,6‐dichlorophenolindophenol titrimetric method described by de Santana et al. [28]. Briefly, 2.0 g of lily bulb samples were homogenized and centrifuged at 10,000 × g for 10 min at 4°C. The supernatant was collected, and the pellet was re‐extracted with 500 *μ*L of extraction buffer, followed by ultrasonic treatment in an ultrasonic cleaner (SB‐4200DTD, SCIENTZ, Ningbo Scientz Biotechnology Co. Ltd., China) at room temperature for 30 min, centrifugation, and oscillation. The supernatants from both extractions were combined for analysis. This protocol also served to assess total phenolic content (TPC) [29] and total flavonoid content (TFC) [30], both reported as mg per gram (mg g^−1^, FW). The determination of GSH content followed Wang et al. [31]. Absorbance was recorded at 412 nm, with results reported as micrograms per gram (*μ*mol g^−1^, FW).

### 2.8. Statistical Assessments

All experiments, including the control and three 1‐MCP treatment groups, were performed with three independent biological batches (*n* = 3), and all indices were measured in triplicate for each batch (technical triplicates). Data analysis was carried out via SPSS 24.0 using one‐way ANOVA, with statistical significance set at *p* < 0.05. Graphical visualization was performed using Origin 2021. Partial correlation analysis was performed separately for the control and 1.5 *μ*L L^−1^ 1‐MCP groups, with storage time set as a controlled covariate. Each correlation coefficient within each group was calculated based on 18 independent destructive‐sampling observations (three biological replicates at each of six sampling time‐points per group), corresponding to a degree of freedom df = 15. All *p* values were corrected for multiple testing using the Benjamini–Hochberg false‐discovery‐rate (FDR) method.

## 3. Results and Discussion

### 3.1. Browning Appearance, Color, and Basic Quality Attributes

Postharvest browning of scale surfaces is the primary factor limiting the commercial quality and market value of Lanzhou lily bulbs during cold storage. The appearance of Lanzhou lilies treated with different 1‐MCP concentrations is shown in Figure 1A. Browning occurred on untreated bulbs after 24 days of storage. Slight browning appeared in the 1.0 *μ*L L^−1^ 1‐MCP group following 36 days of storage, whereas the 1.5 and 2.0 *μ*L L^−1^ cohorts showed only mild yellowing at 48 days and no noticeable browning throughout 60 days. Compared with the treated groups, the control showed accelerated browning and quality decline. Figure 1B showed that at Day 60, the L* value of the 1.5 *μ*L L^−1^ group (69.66, the highest) reflected a 4.44% increase relative to the control. The control group′s a* value remained the highest, significantly exceeding that of the treated groups from Day 36 (*p* < 0.05) (Figure 1C). Regarding b* values, the minimum measurements were recorded in the 1.5 and 2.0 *μ*L L^−1^ groups (Figure 1D). From Day 24 onward, the BD of the control group was markedly higher than that of the treated groups (*p* < 0.05). On Day 60, BDs for the 1.0, 1.5, and 2.0 *μ*L L^−1^ groups were 14.5, 12.24, and 13.15, respectively; these values represented reductions of 15.35%, 28.55%, and 23.23% relative to the control (Figure 1E). These phenotypic differences suggested that 1‐MCP effectively delayed peel discoloration and browning progression in lily bulbs in a dose‐dependent manner within a suitable concentration range. The superior color retention in the 1.5 *μ*L L^−1^ group further suggested that moderate 1‐MCP treatment sufficiently suppressed senescence‐related color deterioration, whereas a relatively low concentration exerted limited protective effects.

**Figure 1:**
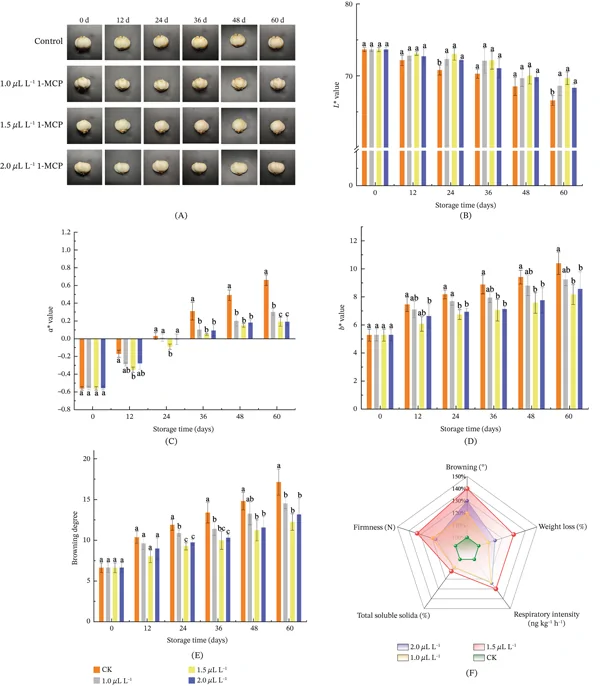
Effects of 1‐MCP treatment on (A) appearance, (B) L* value, (C) a* value, (D) b* value, and (E) browning degree of Lanzhou lily bulbs during storage for 60 days at 4.0^°^C ± 0.5^°^C, as well as radar chart for comprehensive quality evaluation at the end of storage (F). Vertical bars denote mean ± standard deviations for three replicates. Different lowercase letters represent statistically significant differences (*p* < 0.05) at each time point.

Changes in postharvest respiration rate directly mirror the metabolic intensity and senescence progression of plant tissues. As tissue senescence advances, it directly induces and accelerates the browning process in lily bulbs. The respiration rate of Lanzhou lily bulbs declined gradually until Day 48 of storage and began a slow rise thereafter. On Day 48, the rate of the 1.5 *μ*L L^−1^ group reached 14.17 mg CO_2_ kg^−1^ h^−1^, a value markedly lower than that of the control group (*p* < 0.05), indicating suppressed metabolic activity (Table 1). The reduced respiration intensity suggested that optimal 1‐MCP treatment effectively alleviated excessive metabolic consumption, thereby slowing the senescence process of lily bulb tissues.

**Table 1:** Effects of 1‐MCP treatment on respiration rate, firmness, weight loss rate, and total soluble solids of Lanzhou lily bulbs during storage for 60 days at 4.0^°^C ± 0.5^°^C.

Storage time (day)	Treatment (*μ*L L^−1^)	Respiration rate (mg CO_2_ kg^−1^ h^−1^)	Firmness (*N*)	Weight loss (%)	Total soluble solids (°Brix)
0	Untreated	24.90 ± 0.88	29.90 ± 0.19	0.00 ± 0.00	22.67 ± 0.31

12	Control	19.06 ± 0.42^a^	24.87 ± 0.88^a^	1.74 ± 0.11^a^	23.87 ± 0.42^a^
	1.0	18.09 ± 0.26^a^	26.95 ± 0.42^a^	1.63 ± 0.09^ab^	23.74 ± 0.76^a^
	1.5	18.05 ± 0.94^a^	27.53 ± 0.53^a^	1.45 ± 0.11^b^	23.87 ± 1.70^a^
	2.0	18.85 ± 0.51^a^	27.68 ± 0.80^a^	1.55 ± 0.08^b^	23.80 ± 0.92^a^

24	Control	15.18 ± 1.00^a^	22.60 ± 0.82^b^	3.61 ± 0.42^a^	23.47 ± 0.31^a^
	1.0	14.39 ± 1.36^a^	25.39 ± 1.16^a^	3.40 ± 0.63^a^	23.20 ± 0.92^a^
	1.5	14.12 ± 0.93^a^	27.29 ± 0.97^a^	3.01 ± 0.13^a^	24.07 ± 0.95^a^
	2.0	14.45 ± 1.12^a^	26.62 ± 1.33^a^	3.60 ± 0.23^a^	23.07 ± 0.80^a^

36	Control	15.72 ± 0.40^a^	20.33 ± 1.37^b^	5.21 ± 0.31^a^	22.80 ± 0.72^a^
	1.0	14.27 ± 1.08^b^	24.96 ± 1.81^a^	4.65 ± 0.57^a^	23.00 ± 0.87^a^
	1.5	13.86 ± 0.39^b^	26.61 ± 0.66^a^	4.25 ± 0.34^a^	23.40 ± 0.20^a^
	2.0	13.91 ± 0.83^b^	25.91 ± 0.89^a^	4.35 ± 0.78^a^	22.93 ± 0.83^a^

48	Control	17.06 ± 0.86^a^	20.00 ± 0.88^b^	8.35 ± 0.37^a^	21.33 ± 0.64^b^
	1.0	14.31 ± 1.01^b^	23.85 ± 0.49^a^	7.48 ± 0.83^ab^	22.67 ± 0.81^ab^
	1.5	14.17 ± 0.39^b^	25.18 ± 0.70^a^	6.34 ± 0.45^c^	23.13 ± 0.64^a^
	2.0	14.37 ± 0.56^b^	24.44 ± 1.26^a^	6.73 ± 0.53^bc^	22.40 ± 0.80^ab^

60	Control	18.77 ± 0.83^a^	19.04 ± 0.92^c^	10.39 ± 0.46^a^	20.33 ± 0.46^b^
	1.0	15.26 ± 0.53^b^	20.82 ± 0.51^b^	9.66 ± 1.15^a^	22.00 ± 0.72^a^
	1.5	14.39 ± 0.55^b^	25.35 ± 0.95^a^	7.99 ± 0.74^ab^	22.80 ± 0.69^a^
	2.0	15.09 ± 0.78^b^	22.21 ± 0.76^b^	9.11 ± 0.71^b^	21.87 ± 0.70^a^

*Note:* Numerical values in the treatment column represent 1‐MCP concentration (unit: *μ*L L^−1^); untreated and control are nonconcentration treatment groups. Data are expressed as mean ± standard deviations for three replicates. Different lowercase letters represent statistically significant differences (*p* < 0.05) at each time point.

Postharvest water loss is the main cause of firmness reduction in Lanzhou lily bulbs, with tissue firmness closely related to the integrity of cell structure and the maintenance of cellular turgor pressure [32]. Excessive cellular water loss directly damages the stability of cell membranes and cellular compartmentalization, triggering contact between phenolic substrates and oxidases, and thus accelerating the browning of bulb scales [33]. Application of 1‐MCP markedly improved firmness (*p* < 0.05), and starting from Day 24, firmness in the control bulbs was notably lower than that of the treated bulbs. By Day 60, the 1.5 *μ*L L^−1^ group exhibited the maximum firmness of 25.35 N. On Day 60, the weight loss rate of the control group reached 10.39%, whereas those of the 1.0, 1.5, and 2.0 *μ*L L^−1^ groups were 9.66%, 7.99%, and 9.11%, respectively. These results suggested that 1‐MCP retarded postharvest water loss and maintained cell turgor, which collectively preserved tissue firmness and structural integrity, thereby indirectly inhibiting browning occurrence.

The change in TSS content is closely correlated with the senescence process and browning of bulbs during postharvest storage [34]. Lily bulb TSS content increased initially and then declined, peaking on Day 24. The initial increase in TSS may be attributed to starch hydrolysis into soluble sugars during early storage, whereas the subsequent decline likely resulted from respiratory consumption of sugars as senescence progressed. The lowest TSS content (20.33°Brix) was observed in the control bulbs, which was considerably lower than that in all treated bulbs (*p* < 0.05) on the 60th day. This result is in good agreement with the variation trend of BD in Lanzhou lily bulbs. These results suggested that 1‐MCP treatment delayed the depletion of soluble solids, thus maintaining better physiological status and commercial quality of lily bulbs during cold storage.

Radar chart analysis was further performed to comprehensively assess the preservation performance of different 1‐MCP treatments on Lanzhou lily bulbs at the end of 60‐day cold storage, based on five key quality indicators (Figure 1F). As shown in the radar chart, all 1‐MCP‐treated groups exhibited a markedly larger polygon area than the control group, verifying the positive effect of 1‐MCP on maintaining the comprehensive postharvest quality of lily bulbs. Notably, the 1.5 *μ*L L^−1^ 1‐MCP treatment presented the largest polygon area, with optimal performance in all five core indicators, which aligned well with the results of individual indicator analysis. The superior preservation efficacy observed at 1.5 *μ*L L^−1^ relative to the 2.0 *μ*L L^−1^ group points to a nonlinear response rather than a simple concentration‐dependent pattern. One plausible explanation is that, given 1‐MCP functions as a competitive ethylene‐receptor inhibitor, receptor‐binding sites in lily bulb tissues may become nearly saturated at concentrations around 1.5 *μ*L L^−1^, so that additional 1‐MCP delivers no further preservation‐related benefits. A further possibility is that, despite the general safety of 1‐MCP in postharvest handling, supraoptimal concentrations may impose mild physiological stress on bulb tissues, triggering compensatory metabolic responses that partially counteract its protective effects. In addition, we cannot fully exclude treatment variability or other unknown biological factors as potential contributors to this dose‐response pattern. Nevertheless, the consistent advantage of the 1.5 *μ*L L^−1^ group across multiple independent quality indicators suggests this nonlinear response is predominantly biologically driven, rather than stemming from experimental artifacts. This observation underscores the importance of commodity‐specific dosage optimization and demonstrates that ethylene‐inhibitor effects should not be presumed to follow linear dose relationships. This synchronized deterioration in visual quality, texture (firmness), and basic metabolism (respiration, weight loss, and TSS) underscores that browning is not a superficial change but a symptom of systemic senescence.

### 3.2. Membrane Integrity and Oxidative Damage Markers

The cell membrane system is the structural basis for maintaining the physiological activity and postharvest quality of plants, and its stability directly determines the cellular compartmentalization function [35]. To visually verify the effect of 1‐MCP on the membrane structure integrity of Lanzhou lily bulb scales, the three‐dimensional morphology of scale epidermal cells and cross‐sectional tissues was observed by SEM (Figure 2). SEM analysis indicated that on Day 0 (harvest day), the surface of the scale was complete and continuous, with clear cell contours and very little damage, suggesting an intact cell structure. The control bulbs and all treated bulbs (after 60 days of storage) exhibited different degrees of wrinkles and damage, but the degree of damage was different. The scale surface of the control group was rough and uneven, showing not only fine damage but also cell fragments and intracellular material leakage; although the 1.0 and 2.0 *μ*L L^−1^ groups had obvious structural collapse and local cracking, there was no large‐scale disintegration; conversely, the 1.5 *μ*L L^−1^ group′s scale morphology was basically stable with no significant change. In addition, the cross‐cut surface of the 0‐day lily scales showed that the cell membrane boundaries were clear, the starch particles were abundant, and the tissue arrangement was regular; after storage, the control group had severe damage to cell membranes, and the distribution of cells had also become disorderly. The 1.0 *μ*L L^−1^ group showed localized membrane damage and reduced membrane integrity, though its starch grains remained regular and undamaged. Severe epidermal collapse occurred in the 2.0 *μ*L L^−1^ group, but its cell morphology was relatively preserved with only slight boundary blurring and largely intact starch grains. In contrast, the 1.5 *μ*L L^−1^ group maintained well‐preserved cellular morphology, distinct membrane boundaries, and organized architecture with minimal shrinkage and damage. Its starch grains were ellipsoidal, intact, uniform, regularly arranged, and evenly distributed within the stable cell membranes. These microstructural observations provide suggestive visual evidence that 1‐MCP treatment helped preserve cellular and subcellular boundaries. Such maintained compartmentalization may contribute to limiting contact between phenolic substrates and oxidative enzymes such as PPO, which could mitigate the initiation of enzymatic browning.

**Figure 2:**
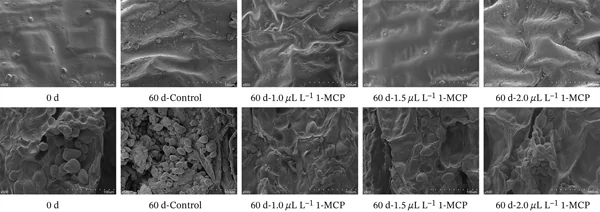
Scanning electron microscope images show the changes in epidermal cells and cross‐sections of Lanzhou lily bulbs during storage. The images depict Lanzhou lily bulbs at harvest (0 days of storage), those treated with 1‐MCP at concentrations of 1.0, 1.5, and 2.0 *μ*L L^−1^, respectively, stored for 60 days.

Membrane lipid peroxidation is the key mechanism of inducing cell membrane damage. This process is primarily activated by LOX‐catalyzed oxidative degradation of linoleic acid and other polyunsaturated fatty acids, which initiates an oxidative cascade reaction that ultimately generates toxic compounds like MDA [36]. The large accumulation of MDA destroys the integrity of the lipid bimolecular layer, which in turn accelerates the peroxidation process of polyunsaturated fatty acids. At the same time, the increase in EL can directly indicate the extent of impairment to the selective permeability of cellular membranes, which is an intuitive indicator for measuring the destruction of membrane integrity [37]. Over the storage period, the EL and MDA levels rose across all experimental groups (Figure 3A,B), whereas LOX activity exhibited a unimodal trend with a peak on storage Day 48 (Figure 3C). Between Days 12 and 60, the 1.5 *μ*L L^−1^ group sustained the lowest EL, with values markedly lower than those recorded for both the control bulbs and all other treated bulbs (*p* < 0.05). The control bulbs exhibited the highest EL, which peaked at 53.46% on Day 60. MDA content in the control bulbs underwent a sharp rise beginning on Day 24 and remained significantly higher than that of all treated bulbs thereafter (*p* < 0.05). As of Day 48, the bulbs treated with 1.5 *μ*L L^−1^ further displayed lower MDA values than those of all other treated bulbs. By the end of storage, MDA content in the 1.5 *μ*L L^−1^‐treated bulbs reached 2.63 *μ*mol g^−1^. In comparison, MDA contents in the 1.0, 2.0 *μ*L L^−1^, and control groups were 3.74, 3.13, and 5.66 *μ*mol g^−1^, respectively. The control group had the highest LOX activity peak (0.96 U g^−1^). Notably, the 1.5 *μ*L L^−1^‐treated bulbs showed a relatively stable LOX catalytic activity, with only a 27% increase over the entire duration of storage.

**Figure 3:**
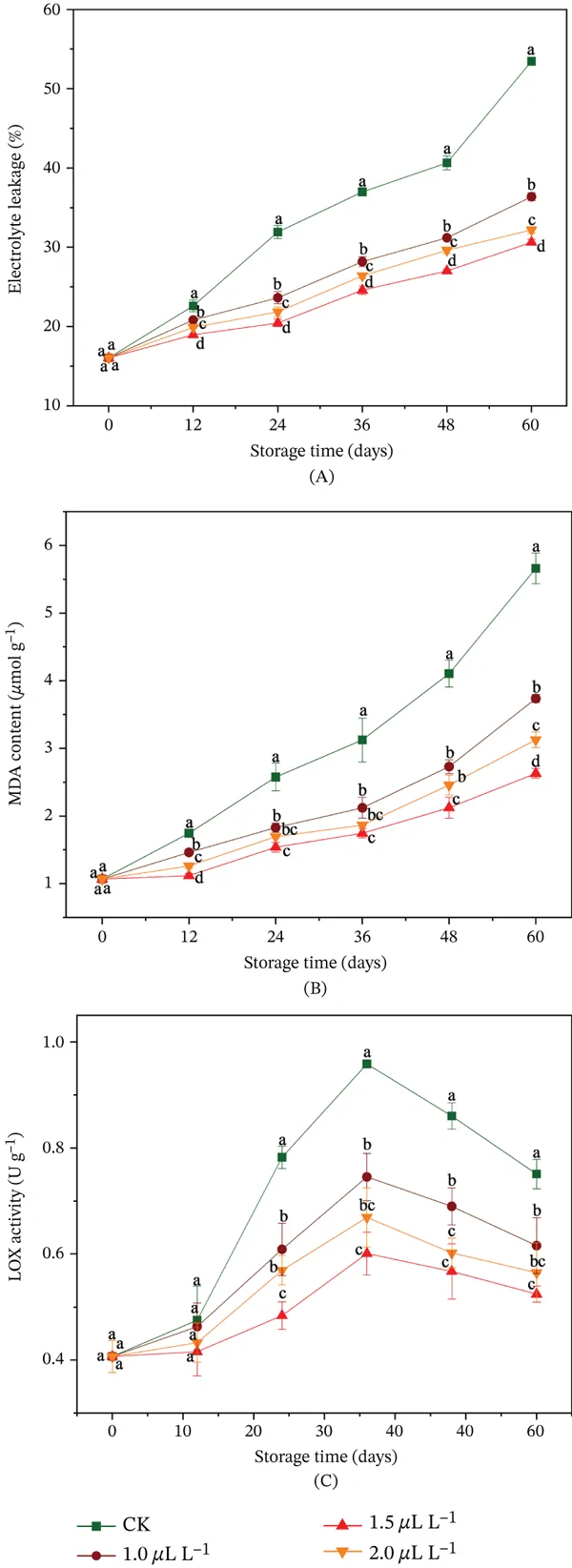
Effects of 1‐MCP treatment on (A) electrolyte leakage, (B) malondialdehyde, and (C) lipoxygenase in Lanzhou lily bulbs during storage at 4.0^°^C ± 0.5^°^C for 60 days. Vertical bars denote mean ± standard deviations for three replicates. Different lowercase letters represent statistically significant differences at each time point (*p* < 0.05).

Our research reveals that treating Lanzhou lily bulbs with 1‐MCP effectively suppresses MDA accumulation and EL elevation. Research has indicated that 1‐MCP is capable of lowering the MDA content of ginger rhizomes [38]. Similar literature reports have also been documented in lotus root [17], purple potato [39], potato [40], and other crops. Furthermore, LOX activity mediates the ripening and senescence of Lanzhou lily bulbs, which is consistent with the research results of Wei et al. [41].

### 3.3. ROS Content and Oxidative Stress

Plant senescence is closely associated with metabolic imbalance in ROS. Large amounts of O_2_^−^·, H_2_O_2_, and other ROS accumulate in plant tissues, and these ROS target membrane lipids, proteins, nucleic acids, and other biological macromolecules, thus inducing oxidative stress [42]. Production rate of O_2_^−^· in lilies initially augmented gradually, followed by a sharp rise during storage (Figure 4A). The O_2_^−^· production rate increased more rapidly in the 1.0 *μ*L L^−1^‐treated and control bulbs. Upon storage completion, the O_2_^−^· production rates in the 1.0, 1.5, and 2.0 *μ*L L^−1^‐treated bulbs were reduced by 12.79%, 27.91%, and 20.93%, respectively, relative to the control group. The trend in H_2_O_2_ content changes mirrored that of the O_2_^−^· production rate (Figure 4B). The control group exhibited a sharp rise in H_2_O_2_ content beginning on Day 48, with the value reaching 2.37 *μ*mol g^−1^ by day 60. Conversely, the 1.0, 1.5, and 2.0 *μ*L L^−1^ groups maintained low H_2_O_2_ levels upon storage completion. Notably, the 1.5 *μ*L L^−1^‐treated bulbs showed the mildest increase.

**Figure 4:**
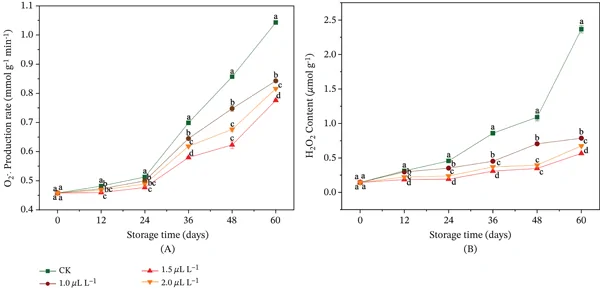
Effects of 1‐MCP treatment on (A) O_2_^−^· production rate and (B) H_2_O_2_ in Lanzhou lily bulbs during storage at 4.0^°^C ± 0.5^°^C for 60 days. Vertical bars denote mean ± standard deviations for three replicates. Different lowercase letters represent statistically significant differences at each time point (*p* < 0.05).

These observations confirm that excessive ROS accumulation is a key driver of oxidative stress and scale browning in postharvest Lanzhou lily bulbs, which is consistent with the conclusion that oxidative stress is closely related to browning in lily bulbs reported by Cheng et al. [1]. Meanwhile, 1‐MCP treatment can effectively enhance the ROS scavenging capacity of bulb tissues, and similar inhibitory effects of 1‐MCP on postharvest ROS accumulation have been widely confirmed in other horticultural crops such as nectarine [43] and apple [44]. To counteract the oxidative damage induced by excessive ROS, Lanzhou lily bulbs activate an endogenous enzymatic antioxidant system composed of multiple functionally coordinated enzymes during cold storage.

### 3.4. Antioxidant System

#### 3.4.1. Enzymatic Antioxidant System

In order to resist the damage caused by ROS, plants themselves activate the enzymatic antioxidant system, and various key enzymes play a role in this system. SOD acts as the primary defensive barrier for antioxidant defense. This enzyme mediates the conversion of O_2_^−^· into H_2_O_2_, and the improvement of SOD activity can effectively reduce the accumulation rate of O_2_^−^· in cells [45]. Additionally, CAT and POD directly participate in the decomposition of H_2_O_2_. Within the AsA‐GSH cycle, the enzyme GR is responsible for reducing GSSG to reduced GSH; the generated GSH reduces dehydroascorbate to AsA under the action of dehydroascorbate reductase. Subsequently, ascorbic acid reduces H_2_O_2_ to water through the catalytic action of APX, itself being oxidized [46]. This chain reaction can not only remove the H_2_O_2_ accumulated in the tissue, but also improve the reduction ability of the cell antioxidant system, thus enhancing the resistance of plants to adversity [47].

Over the course of storage, the activity trends of SOD, CAT, GR, and APX across the four lily bulb groups followed a largely consistent pattern, marked by an initial rise then a subsequent decline (Figure 5A–D). SOD activity peaked on Day 36, with the highest value observed in the 1.5 *μ*L L^−1^‐treated bulbs. For the control bulbs, CAT activity stayed persistently the lowest throughout the storage period. Conversely, all three treated bulb groups′ peak CAT activities were delayed by 12 days relative to the control group, while also being significantly higher (*p* < 0.05). For GR and APX, peak activities occurred on Day 24 in the control, whereas in the 1‐MCP–treated bulbs, the peak activity of both enzymes was postponed to Day 36. Among them, the 1.5 *μ*L L^−1^‐treated bulbs sustained the highest GR and APX activity levels over the full storage period. The 1.5 *μ*L L^−1^‐treated bulbs demonstrated the maximum POD activity, reaching 6.09 U g^−1^ by the 48th day of storage (Figure 5E). Our results are consistent with previous reports showing that 1‐MCP modulates SOD‐gene expression in apple pulp and peel [48] and maintains high antioxidant enzyme activity in pear fruit [49]. Taken together, these observations suggest that 1‐MCP treatment modulates antioxidant enzyme activities in association with delayed senescence of stored Lanzhou lily bulbs.

**Figure 5:**
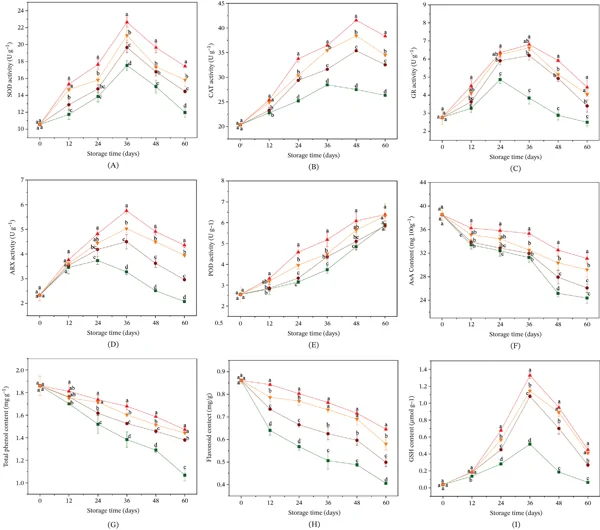
Effects of 1‐MCP treatment on (A) superoxide dismutase, (B) catalase, (C) glutathione reductase, (D) ascorbate peroxidase, (E) peroxidase, (F) ascorbic acid, (G) total phenols, (H) flavonoids, and (I) glutathione in Lanzhou lily bulbs during storage at 4.0^°^C ± 0.5^°^C for 60 days. Vertical bars denote mean ± standard deviations for three replicates. Different lowercase letters represent statistically significant differences at each time point (*p* < 0.05).

More importantly, the synchronized and sustained enhancement of APX and GR activities, key enzymes of the AsA‐GSH cycle, suggests this cycle makes an important contribution to 1‐MCP–mediated protection effects. Unlike the broad upregulation of other enzymes, the specific, delayed peak, and prolonged high activity of APX and GR indicate a tailored defense mechanism against the progressive accumulation of H_2_O_2_, a key oxidant driving membrane damage and browning. Thus, while overall antioxidant capacity is elevated, the AsA‐GSH cycle plays an important role in this antioxidant response [8].

#### 3.4.2. Nonenzymatic Antioxidants and Antioxidant Compounds

In addition to the enzymatic antioxidant system, the nonenzymatic antioxidant system composed of low–molecular‐weight antioxidants is another critical pathway for ROS scavenging and redox homeostasis maintenance in postharvest plant tissues. AsA and GSH are the core nonenzymatic antioxidants in plant cells, which can directly scavenge free radicals, and also act as the key substrates of the AsA‐GSH cycle to sustain the continuous operation of the antioxidant cascade [50]. Meanwhile, phenolic substances such as total phenols and flavonoids can quench ROS and block lipid peroxidation chain reactions via the hydrogen‐donating ability of their phenolic hydroxyl groups; notably, these phenolic compounds are also the core substrates of enzymatic browning, and their oxidative degradation is directly associated with the browning process of Lanzhou lily bulb scales [51].

The contents of AsA, total phenols, and flavonoids in all four experimental groups showed a continuous downward trend during the whole storage period (Figure 5F–H), whereas the GSH content exhibited a trend of initial increase followed by decrease. On Day 60, the contents of AsA, total phenols, and flavonoids in the control group decreased by 9.03 mg 100 g^−1^, 0.79 mg g^−1^, and 0.45 mg g^−1^, respectively, compared with the initial values; in contrast, the decreases in the 1.5 *μ*L L^−1^ group were only 4.03 mg 100 g^−1^, 0.39 mg g^−1^, and 0.21 mg g^−1^. For GSH content, the control group only retained 0.06 *μ*mol g^−1^ at the end of storage, which was 76.09%, 85.67%, and 84.32% lower than those in the 1.0, 1.5, and 2.0 *μ*L L^-1^ groups, respectively (Figure 5I). These results indicate that 1‐MCP treatment can effectively delay the degradation of nonenzymatic antioxidant substances in Lanzhou lily bulbs during cold storage, thus maintaining the nonenzymatic antioxidant capacity of bulb tissues. This is consistent with the findings of Ma et al. [52], who reported that 1‐MCP treatment maintained high levels of AsA and GSH in postharvest broccoli; similar results were also observed in kiwifruit, where 1‐MCP significantly promoted the accumulation of total phenols during cold storage [53].

Plant redox balance is not maintained by a single system, but depends on the synergy of the two major antioxidant systems: enzymatic and nonenzymatic. The AsA‐GSH cycle is the core hub linking these two systems, where the core enzymes APX and GR rely on nonenzymatic antioxidants AsA and GSH as functional substrates to exert their catalytic effects [54]. The effective preservation of AsA and GSH is particularly critical: These compounds are not only major free radical scavengers themselves, but also the core substrates of the AsA‐GSH cycle. Sustained high levels of these compounds ensure a continuous supply of reducing power to support the high activity of APX and GR [55]. This synergy between substrate availability and enzyme activity is essential for the efficient and stable operation of the AsA‐GSH cycle, which is the main pathway for H_2_O_2_ detoxification and the central regulator of cellular redox homeostasis [56]. Our results confirm that 1‐MCP effectively mitigates oxidative damage in Lanzhou lily bulbs by simultaneously activating the enzymatic antioxidant system and delaying the degradation of nonenzymatic antioxidant components. The coordinated operation of this dual antioxidant system maintains cellular redox homeostasis, alleviates oxidative stress and membrane lipid peroxidation, maintains cell compartmentalization, and ultimately inhibits enzymatic browning and preserves the postharvest quality of Lanzhou lily bulbs. This dual antioxidant regulatory mechanism of 1‐MCP has also been widely confirmed in postharvest horticultural crops such as eggplant [57], *Gynura bicolor* [58], and mango [59].

### 3.5. Correlation Analysis of Browning, Quality, Membrane Stability, and Antioxidant System

To further elucidate the regulatory role of 1‐MCP, partial correlation analysis was performed separately for the control and 1.5 *μ*L L^−1^ 1‐MCP groups with storage time set as a controlled covariate (Figure 6). In the control group, significant partial correlations were observed between membrane‐damage–related indicators and quality‐browning parameters. MDA exhibited significant positive partial correlations with L* (*r* = 0.782, *q* < 0.001) and respiration rate (*r* = 0.550, *q* = 0.042), and a significant negative partial correlation with a* (*r* = −0.838, *q* < 0.001). H_2_O_2_ was positively correlated with respiration rate (*r* = 0.724, *q* = 0.005). Regarding the relationship between antioxidants and membrane stability, SOD, CAT, and GSH showed significant negative partial correlations with MDA (SOD: *r* = −0.808, *q* < 0.001; CAT: *r* = −0.773, *q* < 0.001; GSH: *r* = −0.626, *q* = 0.018). Notably, GSH also presented a strong positive partial correlation with LOX (*r* = 0.886, *q* < 0.001), indicating concomitant elevation of GSH content together with LOX activity during storage in control bulbs.

**Figure 6:**
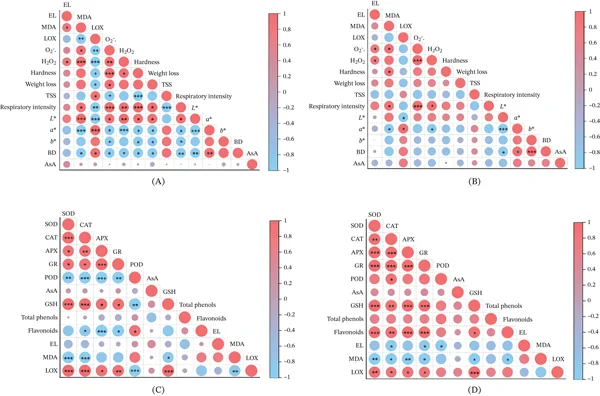
Partial‐correlation analysis of membrane stability and senescence phenotypes in Lanzhou lily bulbs during 60 days of storage at 4.0^°^C ± 0.5^°^C for (A) control and the (B) 1.5 *μ*L L^−1^ group, as well as nonenzymatic antioxidant substances and membrane stability indicators for (C) control, and the (D) 1.5 *μ*L L^−1^ group. Partial correlations were calculated with storage time as a controlled covariate and corrected by the Benjamini–Hochberg FDR method; *q* < 0.05 was considered statistically significant, and *q* < 0.001 was regarded as highly significant.

In the 1.5 *μ*L L^−1^ 1‐MCP–treated group, distinct partial‐correlation features were observed between antioxidant components and membrane‐damage indices. SOD, APX, and GSH displayed significant negative partial correlations with MDA (SOD: *r* = −0.723, *q* = 0.004; APX: *r* = −0.704, *q* = 0.007; GSH: *r* = −0.564, *q* = 0.042). CAT, GR, and flavonoids were also negatively correlated with MDA, whereas CAT, POD, and GR were significantly negatively correlated with EL (all *q* < 0.05). Comparing the two groups, obvious differences existed in partial‐correlation patterns. In the control group, SOD, CAT, and GSH were significantly negatively correlated with MDA, whereas APX and GR, key enzymes of the AsA‐GSH cycle, showed no significant associations with membrane‐damage markers. Following 1‐MCP treatment, APX and GR additionally formed significant negative partial correlations with membrane‐damage indices (MDA and EL), broadening the association network between antioxidant components and membrane stability. Moreover, BD was significantly correlated with multiple membrane‐damage indicators in control bulbs, whereas no significant partial correlation was detected between BD and any membrane‐damage marker under 1‐MCP treatment.

These results suggest that 1‐MCP treatment is associated with maintained cell membrane integrity and delayed postharvest browning and senescence in Lanzhou lily bulbs. The AsA‐GSH cycle appears to be an important component of the overall antioxidant network, potentially contributing to synergistic interplay between enzymatic and nonenzymatic antioxidant systems and reinforcing associations between antioxidant components and membrane stability. In conclusion, the present study indicates that 1‐MCP retards postharvest browning of Lanzhou lily bulbs, with the activation of the AsA‐GSH cycle being one important contributor to endogenous antioxidant defense. As a competitive ethylene‐receptor inhibitor, 1‐MCP is known to interfere with ethylene perception [11]. Although downstream ethylene‐signaling components were not directly measured in the present study, the observed enhancement of antioxidant enzyme activities and nonenzymatic antioxidant contents is consistent with the hypothesis that 1‐MCP treatment triggers a cascade of antioxidant‐defense responses, ultimately mitigating oxidative membrane damage and alleviating postharvest browning.

## 4. Conclusion

A concentration of 1.5 *μ*L L^−1^ 1‐MCP was an effective postharvest treatment for cold‐stored Lanzhou lily bulbs, which significantly inhibited scale browning and senescence and maintained the storage quality and commercial value of the product. The preservation effect of 1‐MCP was associated with enhanced antioxidant defense responses, particularly the AsA‐GSH cycle. Although the upstream ethylene‐related signaling events were not directly investigated, our results demonstrated that 1‐MCP treatment elevated APX and GR activities, maintained the contents of key metabolites (AsA and GSH), and improved ROS scavenging capacity. These responses contributed to reduced membrane lipid peroxidation, preserved membrane integrity, and ultimately alleviated enzymatic browning in Lanzhou lily bulbs.

## Author Contributions

Jiajia Su: writing—original draft, writing—review and editing, methodology, software, investigation, conceptualization. Xiaokun Qiu: writing—review and editing, methodology, investigation, software, validation. Lihua Duan: formal analysis, data curation. Jiaxuan Zheng: data curation, resources. Ping Li: project administration, resources. Runlei Kou: project administration, funding acquisition. Xia Liu: writing—review and editing, project administration, funding acquisition. Qingbin Guo: writing—review and editing, supervision.

## Funding

This study was supported by the Agriculture and Rural Affairs “Small Team” Aid Project in Urumqi (6202512184) and the Taishan Industrial Experts Program (25012712).

## Conflicts of Interest

Ping Li and Runlei Kou are affiliated with Huanxian Food Technology Co. Ltd., Shandong 264402, China. This research was financially supported by Huanxian Food Technology Co. Ltd. The authors declare that the funding sponsor had no role in the study design, data collection, analysis and interpretation, manuscript writing, or the decision to submit the article for publication. All experimental results and conclusions are based on objective data and have not been influenced by commercial interests. All authors confirm that there are no other relevant financial or nonfinancial competing interests to disclose.

## Data Availability

The data that support the findings of this study are available from the corresponding authors upon reasonable request.
